# Dosimetric evaluation of the feasibility of stereotactic body radiotherapy for primary lung cancer with lobe-specific selective elective nodal irradiation

**DOI:** 10.1093/jrr/rrv067

**Published:** 2015-11-12

**Authors:** Tetsuya Komatsu, Etsuo Kunieda, Tadashi Kitahara, Takeshi Akiba, Ryuta Nagao, Tsuyoshi Fukuzawa

**Affiliations:** Department of Radiation Oncology, Tokai University School of Medicine

**Keywords:** stereotactic body radiotherapy, elective nodal irradiation, primary lung cancer, dose–volume histogram

## Abstract

More than 10% of all patients treated with stereotactic body radiotherapy (SBRT) for primary lung cancer develop regional lymph node recurrence. We evaluated the dosimetric feasibility of SBRT with lobe-specific selective elective nodal irradiation (ENI) on dose–volume histograms. A total of 21 patients were treated with SBRT for Stage I primary lung cancer between January 2010 and June 2012 at our institution. The extents of lobe-specific selective ENI fields were determined with reference to prior surgical reports. The ENI fields included lymph node stations (LNS) 3 + 4 + 11 for the right upper lobe tumors, LNS 7 + 11 for the right middle or lower lobe tumors, LNS 5 + 11 for the left upper lobe tumors, and LNS 7 + 11 for the left lower lobe tumors. A composite plan was generated by combining the ENI plan and the SBRT plan and recalculating for biologically equivalent doses of 2 Gy per fraction, using a linear quadratic model. The V_20_ of the lung, D_1cm^**3**^_ of the spinal cord, D_1cm^**3**^_ and D_10cm^**3**^_ of the esophagus and D_10cm^**3**^_ of the tracheobronchial wall were evaluated. Of the 21 patients, nine patients (43%) could not fulfill the dose constraints. In all these patients, the distance between the planning target volume (PTV) of ENI (PTVeni) and the PTV of SBRT (PTVsrt) was ≤2.0 cm. Of the three patients who developed regional metastasis, two patients had isolated lymph node failure, and the lymph node metastasis was included within the ENI field. When the distance between the PTVeni and PTVsrt is >2.0 cm, SBRT with selective ENI may therefore dosimetrically feasible.

## INTRODUCTION

Stage I non–small cell lung cancer (NSCLC) is a potentially curable disease, with the standard treatment being lobectomy with systemic mediastinal lymph node dissection. Unfortunately, up to 25% of patients with Stage I NSCLC are not candidates for lobectomy because of medical problems. Stereotactic body radiotherapy (SBRT) is an alternative treatment option for these patients [1, 2]. SBRT delivers a high biologically effective dose to the tumor, achieving excellent local control and survival rates. Although SBRT has been shown to decrease procedural morbidity and mortality, it has also been associated with higher regional recurrence than that of standard surgical treatment [1].

According to a recent systemic review [3], 10% of all patients treated with SBRT develop regional lymph node recurrence as a pattern of failure. Moreover, the 5-year cumulative nodal failure rate is ∼15% [4]. Although positron emission tomography (PET) scanning with radiolabeled ^18^F-2-fluoro-deoxy-D-glucose (FDG) imaging is a useful diagnostic modality for detecting lymph node metastasis, its ability to detect lymph node metastasis ≤1 cm or lymph node metastasis from adenocarcinoma is limited [5].

On the other hand, a previous surgical study demonstrated the incidence of lymph node metastasis to be associated with the histological type or primary tumor size, and that regardless of the histological type, lymph node failure was not negligible in cases with a primary tumor size ≥2 cm [6]. Therefore, we speculated that elective nodal irradiation (ENI) might be beneficial for select patients with potential lymph node metastasis. The traditional ENI field includes the entire mediastinum. However, the incidence of potential N3 metastasis in patients diagnosed with clinically negative node (cN0) disease is extremely rare, and it has been previously shown to be as low as 0.8% [6]. In addition, surgical research on patients with cN0 disease has revealed the frequency and station of lymph node metastases in relation to the location of the primary tumor. Some thoracic surgeons have performed selective lymph node dissection on patients with cN0 disease and obtained encouraging results [7–9]. Therefore, ENI with a selective field according to the primary location (selective ENI) may be beneficial for some select patients with cN0 disease. On the other hand, performing ENI may compromise the safety of the patient. A previous study reported severe adverse effects caused by SBRT to tumors located within 2 cm of the bronchial tree [10, 11]. An increase in the radiation dose to the lung may also induce unacceptable lung toxicity. Thus, the feasibility of additional selective ENI must be dosimetrically evaluated in order to develop this new approach. In the present study, we assessed the dosimetric feasibility of ENI in addition to SBRT by evaluating the parametric changes in the critical organs, using computed tomography (CT) of patients previously treated with SBRT.

## MATERIALS AND METHODS

### Patients

The present study was approved by the Institutional Review Board of our hospital (No. 13R-091). The patients included in this study had clinically diagnosed Stage I primary lung cancer, as defined by the Union International Contre le Cancer (UICC). Between January 2010 and June 2012, 24 patients were treated with SBRT for Stage I primary lung cancer in our institution. Of these patients, three were excluded from the study because they were treated with a reduced radiation dose that was different from that of our protocol (one patient; 6.5 Gy × 7 fractions, two patients; 6 Gy × 10 fractions) due to a violation in the dose constraints of SBRT for at-risk organs. Data from 21 patients were available for this study. The patient characteristics are shown in Table 1.

**Table 1.** Patient characteristics

Characteristics	
Gender (*n*)	
Male	2 (10%)
Female	19 (90%)
Age (years old)	
median	76
range	56–84
ECOG PS (*n*)	
0	16 (76%)
1	4 (19%)
2	1 (5%)
Histology (*n*)	
Adenocarcinoma	9 (43%)
Squamous cell carcinoma	4 (19%)
unknown	8 (38%)
Tumor size (cm)	
median	2.2
range	1.1–4.4
Tumor site (*n*)	
Right upper lobe	9 (42%)
Right middle lobe	2 (10%)
Right lower lobe	5 (24%)
Left upper lobe	3 (14%)
Left lower lobe	2 (10%)
Dose fractionation (*n*)	
48 Gy/4fr	12 (57%)
50 Gy/5fr	7 (33%)
56 Gy/7fr	2 (10%)
Staging by FDG PET-CT(*n*)	
Yes	11 (53%)
No	10 (47%)
SUV_max_ (value)	
Median	8.5
Range	2.9–13.8

CT = computed tomography, ECOG PS = Eastern Cooperative Oncology Group performance status, FDG = ^18^F-fluorodeoxyglucose, PET = positron emission tomography, SUV _max_ = maximum standardized uptake value.

### SBRT planning and procedure

The patients were fixed in the supine position on a body support immobilization system (Engineering System, Nagano, Japan) with the upper extremities raised using a vacuum pillow for the dorsal aspect of the thorax and a thermoplastic shell for the ventral aspect of the thorax. Tumor movement was fluoroscopically measured prior to treatment planning. An abdominal compression device, equipped with a body support system to reduce respiratory motion, was used when the tumor moved more than 1 cm due to respiration. Serial CT images for treatment planning were acquired with 2-mm slice thickness in the target area and 5-mm in the remaining area, using a 4-row multi-detector CT scanner (HiSpeed NX/I GE Medical Systems, Milwaukee, WI), from the neck to the upper abdomen. The gross tumor volume (GTV) was delineated as the visible tumor at the lung window setting (window width 800 Hounsfield units (HU) and window level –600 HU), using a 3D radiation treatment planning system (Eclipse, Varian Associates, Palo Alto, CA). Two-phase CT images, which consisted of an inspiratory breath-holding phase and an expiratory breath-holding phase, were obtained to determine the range of tumor movement, and a long-scan-time CT with an 8-s scan time in free breathing was also obtained to determine the trajectory of tumor movement. GTVs were contoured on each of the three images to generate the internal GTV from their fusion. The clinical target volume (CTV) for SBRT (CTVsrt) was defined as the internal GTV plus an additional 5–8 mm margin, and the planning target volume (PTV) for SBRT (PTVsrt) was created by a 3-mm expansion of the CTVsrt in all directions. The dose was prescribed at the center of the PTV, and the PTV was covered with an isodose line between 80 and 90% of the prescription dose. The leaf margins were adjusted in an effort to improve conformity. The radiation doses were calculated using an analytical anisotropic algorithm (AAA) implemented in Eclipse 10.0.28 with heterogeneity correction. The calculation grid size was 0.25 × 0.25 × 0.25 cm. All patients were treated using 6-MV X-rays with non-coplanar static fields (ranging from 7 to 9 fields). Radiation treatment was then performed after image verification with 2D matching of the kilovoltage planar image and 3D matching of cone-beam CT acquired with the Varian on-board imaging (OBI) system equipped at the linear accelerator (CLINAC 21EX, Varian Medical Systems, Palo Alto, CA). The dose constraints of our protocol were determined based on the Japan Clinical Oncology Group (JCOG) Phase II clinical trial for Stage IA NSCLC (JCOG 0403 protocol) in consideration of other reports [12–14]. Table 2 shows the dose constraints used at our institution. Protocol 1 was of the utmost priority. When treatment planning could not fulfill the Protocol 1 dose constraint of SBRT, then Protocol 2 was adopted. If the recalculated plan could not fulfill Protocol 2, then the patient was judged ineligible for SBRT.

**Table 2.** Dose constraints of stereotactic body radiotherapy

		Protocol 1	Protocol 2
		Dose prescription	
		12 Gy × 4 fractions or 10 Gy × 5 fractions	8 Gy × 7 fractions
Dose constraint			
Organ	Volume	Total dose (dose per fraction)	
Esophagus	1 cm^3^	<38 Gy (7 Gy)	<44 Gy (7 Gy)
Esophagus	10 cm^3^	<33 Gy	<37 Gy
Trachea and the main bronchus	1 cm^3^	<38 Gy	<44 Gy
Trachea and the main bronchus	10 cm^3^	<33 Gy	<37 Gy
Spinal cord	Max dose	<25 Gy (5 Gy)	<25 Gy (5 Gy)
Lung	Mean dose	<18 Gy	<18 Gy
	Volume <25%	12 Gy	14 Gy
Brachial plexus	Max dose	<42 Gy	<50 Gy

### Producing selective ENI fields

A search using the PubMed electronic database was conducted using the words ‘non–small cell lung cancer’, ‘lymph node metastasis’ and ‘Stage I’ to determine the optimal selective ENI fields. The criteria by which we selected articles were: a sufficient number of cases, pathological findings, and reference to the frequency and location of lymph node metastasis according to primary sites. We found 10 articles that met the above criteria and provided useful information for our study. A summary of these articles is as follows.

- In surgery for cN0 lung cancer, hilar lymph node metastasis plus mediastinal lymph node metastasis is the most common pattern, with a frequency of 39%, followed by hilar lymph node metastasis alone (17%) and mediastinal lymph node metastasis alone (17%). The frequency of intrapulmonary lymph node metastasis is low (7.4%) [15].
- Oda *et al.* [6] performed lobectomy with systemic mediastinal lymph node dissection for cN0 disease and reported that 78%, 8%, 13% and 0.8% of those patients were pN0, 1, 2 and 3, respectively.
- It was found that 72–73% of micrometastases cases demonstrated metastasis to a single station, in which the range of metastasis was localized, and the prognosis was better than that of metastasis to multiple stations [16].
- In several surgical reports [17–23], a relationship was observed between the location of the primary tumor and the lymph node stations likely to be metastasized. These reports are summarized in Table 3. Because the nomenclature for lymph node stations in the above studies was according to the standard lymph node map proposed by Naruke *et al.* [24], we also used the same nomenclature. (i.e. #1: highest mediastinal nodes, #2: upper paratracheal nodes, #3: prevascular and retrotracheal nodes, #4: lower paratracheal nodes, #5: subaortic nodes (aortic–pulmonary window), #6: para-aortic nodes, #7: subcarinal nodes, #8: paraesophageal nodes, #10: hilar nodes and #11: interlobar nodes).

**Table 3.** Summary of the literature regarding the frequency of lymph node metastasis

Author (reference)	Watanabe *et al.* [17]	Naruke *et al.* [18]	Kotoulas *et al.* [19]	Cerfolio *et al.* [20]	*Asamura et al.* [21]	*Ichinose et al.* [22]	*Turna et al.* [23]
Number of Patients	124	1815	557	954	166	402	280
Objects	Operated on N2 cases	Operated on all cases	Operated on all N2 cases	Operated on all cases	Operated on *single N2 cases* (94 patients)	Operated on *single N2 cases* (209 patients)	Operated on *single N1, N2 cases*(216 patients)
Location	Major metastatic station (frequency)						
RUL	#3 (73%) #2 (40%)	#3 (12.3%) #4 (8%)	#4 (76%) #3 (53%)	#4R (23%) #2R (17%)	#3 (38.9%) #4 (16.7%)	#3 (59%) #4 (23%)	#11 (16.7%) #4 (13.3%)
RML	#7 (69%) #3 (47%)	#3 #7	#4 (50%) #7 (50%)	#4R (8%) #7 (6%)	#7 (25%) #3 (12.5%)	#7 (62%) #3 (16%)	none
RLL		#7 (13.7%)	#8 (58%) #7 (42%)	#4R (15%) #7 (14%)	#7 (24.4%) #3 (19.5%)		#11 (17%) #7 (15.2%)
LUL	#5 (71%) #6 (43%)	#5 (12.3%) #6 (6.7%)	#5 (84%) #3 (26%)	#6 (16%) #5 (14%)	#5 (25%) #7 (11.4%)	#5 (61%) #6 (19%)	#11 (22.4%) #5 (10.4%)
LLL	#8 (50%) #7 (38%)	#7 (11.9%)	#7 (44%) #8 (44%)	#7 (8%) #6 (7%)	#7 (26.3%) #4 (15.8%)	#7 (57%) #4 (17%)	#11 (11.8%) #7 (7.8%) #8 (5.9%)

#1 = superior mediastinal node, #2 = paratracheal node, #3 = pretracheal and retrotracheal node, #4 = tracheobronchial node, #5 = subaortic node, #6 = paraaortic node, #7 = subcarinal node, #8 = paraesophageal node, #11 = interlobar nodes, LLL = left lower lobe, LUL = left upper lobe, RLL = right lower lobe, RML = right middle lobe, RUL = right upper lobe, single N2 case = patients with lymph node metastasis in only one N2 station, single station case = patients with lymph node metastasis in only one station.

Referring to the summary of the above reports on mediastinal metastasis of lung cancer, we set mediastinal ENI fields at lymph node stations with a probability of mediastinal lymph node metastasis of 20% or higher with the aim of inhibiting single station lymph node metastasis. Finally, lobe-specific selective ENI fields were set at the lymph node regions with a 20% or higher frequency of lymph node metastasis according to reports by Asamura and Ichinose *et al.* [21, 22], who investigated single N2 cases, and at those with a 10% or higher frequency according to the report by Turna *et al.* [23], who investigated single N1–N2 cases. (These three reports investigated single station cases.) The fields were defined as follows, according to the primary lesions:

right upper lobe (RUL): lymph node stations #3, #4, #11

right middle lobe and right lower lobe (RML-RLL): lymph node stations #7, #11

left upper lobe (LUL): lymph node stations #5, #11

left lower lobe (LLL): lymph node stations #7, #11.

The CTVeni, which indicates the CTV of the selective ENI field, was delineated according to the CT atlas of the lymph node stations clearly defined by Chapet *et al.* [25] (based on the American Joint Committee on Cancer (AJCC) lymph node map originally described in a study by Naruke). In their procedure, #10 and #11 were grouped together because it was difficult to differentiate them from each other on axial CT images. The mediastinal lymph node stations were delineated at the soft tissue setting (window width 400 HU and window level +20 HU), and hilar node stations were delineated using a window level of 350 HU with a width of 2200 HU. The PTVeni, which indicates the PTV of the selective ENI field, was created by a 5-mm expansion of the CTVeni in all directions. A 5-mm margin is appropriate according to recent verification systems [26]. Although the beam arrangement was basically anteroposterior opposing fields, oblique opposing fields with a 10–20° angle were adopted when an overlap between the PTVeni and PTVsrt or the spinal cord could be excluded from the treatment field with an oblique angle. The optimal radiation dose for ENI was determined, based on the findings of Kepta's study [27]. The prescription dose was 40 Gy in 20 fractions at the center of the PTVeni.

In this study, we selected the spinal cord, lung, esophagus and tracheobronchial wall as the organs at risk (OARs). Contouring of these organs was performed according to the atlas presented by Kong *et al.* [28]. The tracheobronchial wall was contoured with a 2-mm thickness from the level of the lung apex to the origin of the segmental bronchus ipsilaterally and to the origin of the lobar bronchus contralaterally. The spinal cord was delineated as the entire space within the bone canal.

### Total evaluation

To evaluate the total biological dose of the two treatment plans comprising SBRT and the additional ENI plan, we used biologically equivalent doses of 2 Gy per fraction (EQD2) from the linear quadratic (LQ) formula with an α/β of 3 for late-responding tissues (i.e. the lung, esophagus and tracheobronchial wall) and an α/β of 2 for the spinal cord using the following equation for the SBRT plan:

EQD2=N×d× [(d+α/β)/(2Gy +α/β)],

where *N* is the number of fractions of SBRT and *d* is the dose per fraction adopted in SBRT.

We recalculated the SBRT plan into the EQD2 plan by changing the prescription, namely 4 × 12 Gy into 72 × 2 Gy, 5 × 10 Gy into 65 × 2 Gy, and 7 × 8 Gy into 61 × 2 Gy, to evaluate the lung, esophagus and tracheobronchial wall, and also converted 4 × 12 Gy into 84 × 2 Gy, 5 × 10 Gy into 75 × 2 Gy, and 7 × 8 Gy into 70 × 2 Gy, to evaluate the spinal cord. Composite plans were then generated by combining the EQD2 plan of the SBRT and ENI plans, and total dosimetric data for each organ were obtained from these to estimate the feasibility on dose–volume histograms (DVH). The lung as an OAR was defined as the total lung minus the GTV and was estimated from the V_20_, which indicates the percentage of volume that received ≥20 Gy. The esophagus was estimated from the D_1cm^3^_, which indicates the minimal radiation doses for the most irradiated volume of 1 cm^3^, and the D_10cm^3^_, which indicates the minimal radiation doses for the most irradiated volume of 10 cm^3^. The spinal cord was estimated from the D_1cm^3^_**,** and the tracheobronchial wall was estimated from the D_10cm^3^_.

To define the dose constraints of OARs, we searched for studies using the PubMed database regarding radiation-induced toxicity and the tolerance dose for OARs. Although the number of available studies was limited because of sporadic reports, we determined the dose constraints of the composite plan for OARs as follows. The V_20_ of the lung was ≤30% [29, 30]. D_1cm^3^_ of the spinal cord was ≤50 Gy. D_1cm^3^_ and D_10cm^3^_ of the esophagus were ≤70 Gy and ≤60 Gy, respectively [31–33], and D_10cm^3^_ of the tracheobronchial wall was ≤70 Gy [34–36]. Because the evaluation of the planning OAR volume was not always a reliable assessment procedure, we did not use this concept [37].

## RESULTS

### DVH evaluation

**Fig. 1.**
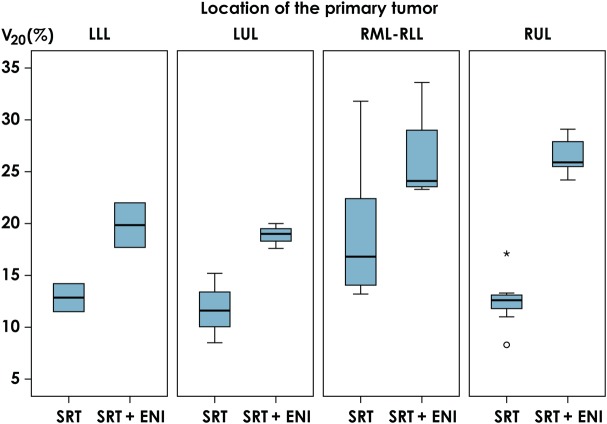
Changes in V_20_ of the lung according to the location of the primary tumor. ENI = elective nodal irradiation, LLL = Left lower lobe, LUL = Left upper lobe, RLL = Right lower lobe, RML = Right middle lobe, RUL = Right upper lobe, SRT = stereotactic body radiotherapy, V_20_ = percentage of the volume that received ≥20 Gy.

**Fig. 2.**
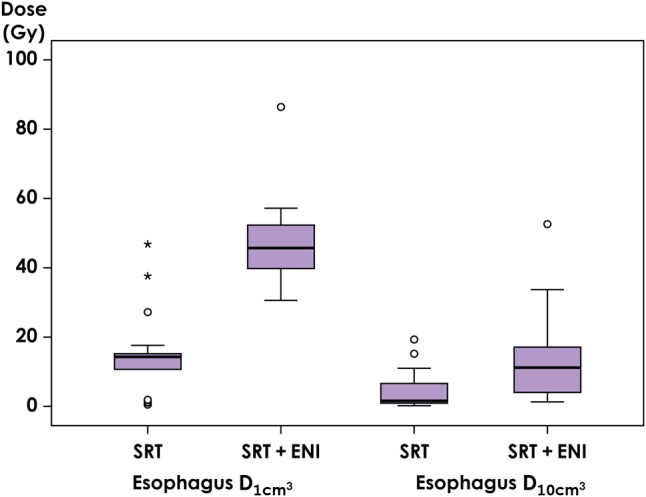
Changes in D_1cm^3^_ and D_10cm^3^_ of the esophagus. D_1cm^3^_ = the minimal radiation doses for the most irradiated volume of 1 cm^3^, D_10cm^3^_ = the minimal radiation doses for the most irradiated volume of 10 cm^3^, ENI = elective nodal irradiation, SRT = stereotactic body radiotherapy.

**Fig. 3.**
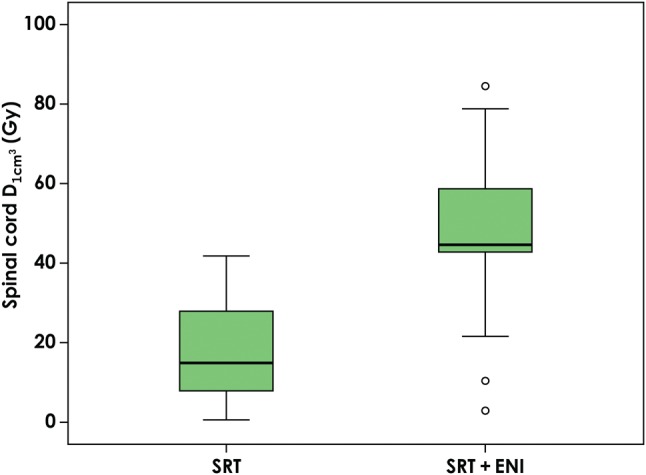
Changes in D_1cm^3^_ of the spinal cord. D_1cm^3^_ = the minimal radiation doses for the most irradiated volume of 1 cm^3^, ENI = elective nodal irradiation, SRT = stereotactic body radiotherapy.

**Fig. 4.**
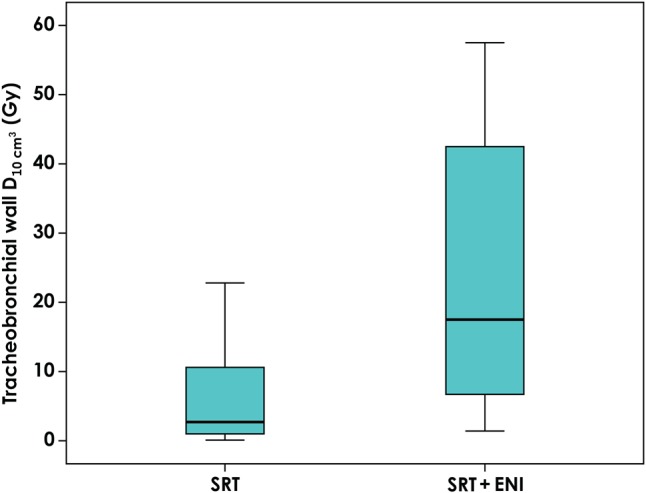
Changes in D_10cm^3^_ of the tracheobronchial wall. D_10cm^3^_ = the minimal radiation doses for the most irradiated volume of 10 cm^3^, ENI = elective nodal irradiation, SRT = stereotactic body radiotherapy.

Of all the 21 patients, nine patients (43%) could not fulfill the dose constraints of the composite plan for the OARs determined in this study. The V_20_ of the lung in all patients increased from a median value of 13.2% (range: 8.3–31.8%) to a median value of 24.4% (range: 17.6–33.6%) after adding the ENI plan. Figure 1 shows changes in the V_20_ according to the primary site. The addition of ENI increased the median V20 from 12.6% (range: 8.3–17.1%) to 25.9% (range: 24.2–29.1%) in patients with RUL primary lesions, from 16.8% (13.2–31.8%) to 24.1% (23.3–33.6%) in those with RML-RLL primary lesions, from 11.6% (8.5–15.2%) to 19.0% (17.6–20.0%) in those with LUL primary lesions, and from 12.8% (11.5–14.2%) to 19.8% (17.7–22.0%) in those with LLL primary lesions. Figure 2 shows an evaluation of the esophagus. The addition of ENI increased the median D_1cm^3^_ from 14.3 Gy (range: 0.5–46.8 Gy) to 45.7 Gy (range: 30.6–86.4 Gy), and the median D_10cm^3^_ from 1.6 Gy (0.2–19.3 Gy) to 11.2 Gy (1.3–52.6 Gy). Both D_1cm^3^_ and D_10cm^3^_ were lower than 60 Gy in most patients, even though ENI was added, while D_1cm^3^_ and D_10cm^3^_ increased to 86.4 and 52.6 Gy, respectively, in one patient. Figure 3 shows the changes in the dosimetric parameters of the spinal cord. The median D_1cm^3^_ increased from 14.9 Gy (range: 0.6–41.8 Gy) to 44.6 Gy (range: 2.9–84.5 Gy). Figure 4 shows changes in the D_10cm^3^_ of the tracheobronchial wall. Although, the addition of ENI increased the D_10cm^3^_, the values were lower than 60 Gy in all patients. Table 4 shows the nine patients who could not fulfill the dose constraints of OAR. The most common cause was the dose to the spinal cord. In 6 of the remaining 12 patients, the distance between two PTVs was ≤2.0 cm. In fact, 60% of patients with a distance between two PTVs of ≤2.0 cm could not fulfill the dose constraint. All patients with a distance between two PTVs >2.0 cm were thus able to fulfill the dose constraints.

**Table 4.** List of nine patients who could not fulfill the dose constraints

No.	Location	Distance	Lung V_20_	Esophagus D_1cm^3^_ (Gy)	Esophagus D_10cm^3^_ (Gy)	Spinal cord D_1cm^3^_	Tracheo-bronchial wall D_10cm^3^_
1	RUL	2.0 cm	24.2%	57.2 Gy	42.5 Gy	***67.3* Gy**	41.6 Gy
2	RUL	1.0 cm	27.3%	***86.4* Gy**	52.6 Gy	***84.5* Gy**	57.5 Gy
3	RML	0 cm	24.1%	48.5 Gy	8.0 Gy	***51.2* Gy**	2.3 Gy
4	RLL	1.4 cm	24.4%	39.8 Gy	4.0 Gy	***59.1* Gy**	10 Gy
5	RLL	0.5 cm	***33.6*%**	44.8 Gy	13.0 Gy	44.6 Gy	6.7 Gy
6	RUL	1.9 cm	25.9%	54.7 Gy	18.3 Gy	***55.5* Gy**	50.8 Gy
7	RUL	1.2 cm	28.3%	45.7 Gy	4.1 Gy	***58.7* Gy**	42.5 Gy
8	RUL	0 cm	29.1%	54.9 Gy	19.2 Gy	***78.8* Gy**	50.1 Gy
9	RUL	1.0 cm	***33.6*%**	52.3 Gy	4.5 Gy	***71.6* Gy**	1.9 Gy

D_1cm^3^_ = the minimal radiation doses for the most irradiated volume of 1 cm^3^, D_10cm^3^_ = the minimal radiation doses for the most irradiated volume of 10 cm^3^, distance = distance between the planning target volume (PTV) for stereotactic body radiotherapy and the PTV for selective elective nodal irradiation field, LLL = left lower lobe, LUL = left upper lobe, RLL = right lower lobe, RML = right middle lobe, RUL = right upper lobe, V_20_ = percentage of the volume that received ≥20 Gy.

### Relationship between the ENI field and the outcomes

The median follow-up time after SBRT was 26 months (range: 3–38 months). The 2-year overall survival rate was 84%, and the 2-year local control rate was 88%. Grade 2 radiation pneumonitis, according to the common terminology criteria for adverse events (CTCAE) version 4.0, was observed in one patient. Local recurrence was observed in two patients and regional lymph node metastasis was observed in three patients. Three patients developed distant metastasis.

Of the three patients who developed regional metastasis, two patients had isolated lymph node failure in a single station as the initial site. The other patient had both hilar node metastasis and local recurrence. Although 11 patients underwent PET-CT before the treatment, none of the three patients with lymph node metastasis did. One of the two patients with isolated lymph node failure had a RUL primary tumor and developed lymph node metastasis in #3 two months after the completion of SBRT. The other patient had a LUL primary tumor and developed lymph node metastasis in #5 four months after the completion of SBRT. The former patient developed further metastasis to the pleura and lung seven months later. In both patients, lymph node metastasis was included within the additional ENI field.

## DISCUSSION

The treatment outcome of conventional radiotherapy for Stage I NSCLC has been poor, with a 5-year overall survival rate of 13−27% [38, 39]. In later studies, the rate of regional lymph node recurrence after irradiation of the primary lesions alone was revealed to be very low (6–8%) [40, 41]. Thus, irradiation to the primary tumor alone at a high dose has become a common treatment strategy. For the past 10 years, a high local control rate (∼90%) has been achieved for Stage I primary lung cancer, using the stereotactic radiosurgery technique [3].

However, even when all patients were staged by FDG-PET, the 5-year regional recurrence rate after SBRT was ∼12.7% [42], and lymph node recurrence was the second cause of disease recurrence after distant metastasis. Because ENI may increase treatment-related toxicities, the selection of patients is important. Senthi *et al.* [42] noted that 83% of the patients with locoregional recurrence did not develop any subsequent distant metastasis. Ohta *et al.* [43] demonstrated that the survival of patients with lymph node micrometastasis with vascular endothelial growth factor (VEGF) overexpression at the primary site was worse than that of patients with lymph node micrometastasis without VEGF overexpression. In addition, the survival of patients with nodal micrometastasis without VEGF overexpression was almost equivalent to that of patients without nodal micrometastasis. Whether a small increase of regional control affects the overall survival or quality of life is unknown, but taking the results of the above studies into consideration, for select patients, ENI may confer a survival benefit after SBRT. A previous study showed that histopathological differentiation and tumor size were related to nodal metastasis in patients with clinical Stage I NSCLC [15]. Oda *et al.* [6] reported that they did not observe lymph node metastasis in patients with adenocarcinoma ≤10 mm in diameter or squamous cell carcinoma ≤20 mm.

Parashar *et al.* [44] estimated the incidental radiation dose to each lymph node station with respect to SBRT. They showed that a clinically significant radiation dose, which is equivalent to a dose of 25 Gy in standard fractionation, was delivered to ∼15% of draining lymph node stations and speculated that the incidental irradiation to lymph node stations might reduce the rate of nodal recurrence. However, Martin *et al.* [45] showed that an incidental irradiation dose of SBRT was insufficient for peripherally located tumors. We were concerned about possible side effects from the addition of ENI and thus performed a planning study. While salvage radiotherapy may be one of the options for lymph node recurrence, a higher radiation dose will be required to eradicate the macroscopic metastatic lesions, and the irradiated lung volume will enlarge. Thus salvage radiotherapy may be more risky for both mediastinal organs and the lung than ENI. Our study showed that we should pay careful attention to the dose to the spinal cord, lung and esophagus, and that the dose to the spinal cord was the most critical factor. To fulfill the dose constraint, the distance between the PTVeni and PTVsrt required more than 2.0 cm. Patients with peripherally located adenocarcinoma or rather large squamous cell carcinoma may thus be suitable for ENI.

There were several limitations associated with this study. One limitation was the use of the LQ model for evaluations. Although Gay *et al.* [46] also calculated the tolerance dose using the LQ model and noted that potential fetal complications from hypofractionated radiotherapy (such as massive hemoptysis, bronchial stenosis and esophageal perforation, which are rarely seen in conventional treatment) may occur in the high dose range. However, as far as radiation pneumonitis was concerned, Borst *et al.* [47, 48] demonstrated that an LQ model with an α/β of 3 was appropriate by clinically comparing the incidence of radiation pneumonitis between conventional radiotherapy and SBRT using the LQ model. To determine whether ENI is effective for selecting the patient, a further clinical trial will be needed after an evaluation of the clinical safety of this treatment. This study will provide fundamental information for further clinical study.

## CONCLUSION

In conclusion, the addition of lobe-specific selective ENI to SBRT for patients with clinical Stage I lung cancer is therefore considered to be feasible on a DVH evaluation, when the distance between the 2 PTVs was more than 2 cm. Further clinical studies are therefore needed to confirm the feasibility of SBRT with ENI.

## FUNDING

Funding to pay the Open Access publication charges for this article was provided by Tokai University.
